# The emerging role of ZDHHC9 in cancer: from protein palmitoylation to tumor progression and immune regulation

**DOI:** 10.3389/fimmu.2026.1867145

**Published:** 2026-07-15

**Authors:** Yi Fu, Ming Ma, Hailong Zhao, Zunyao Xiao

**Affiliations:** 1 Department of General Surgery, The Second Affiliated Hospital of Dalian Medical University, Dalian, Liaoning, China; 2 Department of General Surgery, Huangyuan County People’s Hospital Xining, Xining, Qinghai, China; 3 Department of Gynecology II, The Second Affiliated Hospital of Dalian Medical University, Dalian, Liaoning, China

**Keywords:** cancer progression, immune regulation, palmitoylation, therapeutic targeting, ZDHHC9

## Abstract

Protein S-palmitoylation, and more broadly protein S-acylation when acyl donors other than palmitoyl-CoA are involved, is an emerging post-translational mechanism in cancer that dynamically regulates protein stability, membrane localization, trafficking, and signaling output. Among the zinc finger DHHC-type palmitoyl acyltransferases, ZDHHC9 has attracted increasing attention because of its expanding roles across multiple malignancies. Accumulating evidence indicates that ZDHHC9 is frequently dysregulated in cancer and contributes to tumor progression through palmitoylation-dependent regulation of proteins involved in oncogenic signaling, stress adaptation, metabolic rewiring, and immune evasion. Functionally, ZDHHC9 has been linked to the modulation of substrates such as BiP/GRP78, CD38, STAT1, PD-L1, and PCBP1, thereby influencing unfolded protein response signaling, checkpoint-related immune suppression, ferroptosis-associated pathways, and tumor cell survival. Beyond its tumor-intrinsic effects, ZDHHC9 is increasingly recognized as a regulator of the tumor immune microenvironment, with emerging evidence showing that it can suppress effector CD8+ T-cell responses and reduce sensitivity to immune checkpoint blockade in selected cancer contexts. In this review, we summarize the molecular basis of ZDHHC9, discuss its roles in tumor progression and immune regulation, and highlight current advances and challenges in therapeutic targeting. A deeper understanding of ZDHHC9-dependent palmitoylation networks may support the development of new biomarkers and precision anticancer strategies.

## Highlights

- ZDHHC9-mediated palmitoylation drives tumor growth, stress adaptation, and metabolic rewiring.
- ZDHHC9 promotes immune evasion by regulating PD-L1 signaling and suppressing CD8+ T-cell responses.
- Targeting ZDHHC9 represents a promising strategy to enhance cancer therapy, particularly immunotherapy.

## 1 Introduction

Cancer progression is driven not only by genetic and transcriptional alterations, but also by dynamic post-translational regulatory mechanisms that reshape protein function, signal transduction, and cellular adaptation (1, 2). Among these mechanisms, protein lipidation has emerged as an important yet still underappreciated layer of regulation in tumor biology. In particular, S-palmitoylation, a reversible lipid modification involving the covalent attachment of palmitate to cysteine residues, has attracted increasing attention because of its capacity to control protein stability, membrane association, subcellular trafficking, intermolecular interactions, and signal output (3, 4). Unlike many other lipid modifications, S-palmitoylation is reversible, allowing it to function as a dynamic molecular switch that fine-tunes cellular behavior in response to environmental and intracellular cues. Growing evidence indicates that aberrant palmitoylation contributes to multiple hallmarks of cancer, including sustained proliferative signaling, resistance to cell death, metabolic adaptation, immune escape, and therapeutic resistance.

The enzymatic machinery that controls S-palmitoylation is largely composed of the zinc finger DHHC-type palmitoyl acyltransferase (ZDHHC) family. These enzymes catalyze the transfer of palmitate to substrate proteins and thereby influence a broad spectrum of biological processes (5–7). Although several ZDHHC family members have been implicated in cancer, recent studies suggest that ZDHHC9 is emerging as a particularly interesting regulator at the interface of oncogenic signaling and tumor–microenvironment interactions. Initially studied in the context of developmental disorders and Ras-related signaling, ZDHHC9 has now been linked to a growing number of malignancies, including bladder cancer, pancreatic cancer, gastric cancer, lung adenocarcinoma, breast cancer, colon cancer, prostate cancer, and osteosarcoma (8). This expanding body of work suggests that ZDHHC9 is not merely a housekeeping palmitoyltransferase, but rather a context-responsive regulator whose downstream consequences may vary according to substrate availability, tumor lineage, and immune environment. Mechanistically, ZDHHC9 has been shown to influence cancer biology through the palmitoylation of diverse proteins involved in tumor growth, stress adaptation, immune signaling, and metabolic homeostasis. Recent studies indicate that ZDHHC9-mediated palmitoylation can stabilize or functionally reprogram molecules such as BiP/GRP78, CD38, STAT1, and PD-L1, thereby affecting unfolded protein response signaling, oncogenic survival pathways, immune checkpoint regulation, and tumor–immune crosstalk. In addition, emerging evidence links ZDHHC9 to broader malignant programs, including RAS/MAPK activation, polyamine metabolism, ferroptosis-related pathways, and the establishment of an immunosuppressive tumor microenvironment (9–11). Particularly notable is the increasing recognition that ZDHHC9 may modulate not only tumor cell-intrinsic behavior but also the responsiveness of tumors to immunotherapy, especially immune checkpoint blockade. These findings have substantially broadened the conceptual framework of ZDHHC9 research, shifting it from a substrate-centered biochemical topic toward a more integrative cancer biology perspective.

At the same time, the current literature also indicates that the role of ZDHHC9 in cancer should not be oversimplified. While most available studies support a tumor-promoting function, the biological impact of ZDHHC9 appears to be context-dependent, and may differ across cancer types, molecular backgrounds, substrate repertoires, and microenvironmental states. In some settings, ZDHHC9-associated pathways intersect with oxidative stress control and ferroptosis regulation in ways that suggest more nuanced or even bidirectional functional consequences (12, 13). Moreover, despite rapidly increasing interest in ZDHHC9, many important questions remain unresolved, including how its substrate specificity is determined in different tumors, how it cooperates with other post-translational modifications, and whether its enzymatic activity can be selectively and safely targeted for therapeutic benefit.

In this review, we summarize the emerging evidence for the role of ZDHHC9 in cancer, with a particular focus on how protein palmitoylation links ZDHHC9 to tumor progression and immune regulation. We first outline the molecular and biological basis of ZDHHC9 and the functional consequences of S-palmitoylation. We then discuss its involvement in tumor cell-intrinsic malignant programs, including proliferation, survival signaling, stress adaptation, and metabolic rewiring. Next, we examine the growing evidence connecting ZDHHC9 to immune suppression, immune checkpoint regulation, and immunotherapy response. Finally, we highlight the therapeutic potential of targeting ZDHHC9-dependent palmitoylation networks, as well as the conceptual and translational challenges that need to be addressed in future studies.

## 2 Molecular and biological basis of ZDHHC9

### 2.1 Conserved structural and functional features of the ZDHHC family

The ZDHHC family comprises a group of membrane-associated protein S-acyltransferases that catalyze the attachment of fatty acyl chains to cysteine residues on substrate proteins (6, 7, 14). A defining feature shared by all family members is the conserved Asp-His-His-Cys (DHHC) catalytic motif, which is essential for enzymatic activity. Most ZDHHC enzymes contain multiple transmembrane domains that anchor them to intracellular membranes, including the endoplasmic reticulum, Golgi apparatus, endosomes, and plasma membrane (15–17). This membrane localization is functionally important because S-acylation is often spatially regulated and can determine where substrate proteins acquire membrane affinity, signaling competence, or trafficking capacity. Functionally, ZDHHC enzymes regulate diverse biological processes by controlling substrate localization, stability, trafficking, protein–protein interactions, and signaling output (18, 19). Despite sharing a conserved catalytic mechanism, individual ZDHHC family members differ in their subcellular distribution, accessory protein requirements, substrate preference, and tissue-specific functions. These differences help explain why distinct ZDHHC enzymes can produce nonredundant biological effects even though they catalyze a chemically similar modification. Within this broader family context, ZDHHC9 retains the conserved DHHC-dependent S-acyltransferase activity but is distinguished by its prominent Golgi localization, functional dependence on the accessory protein GCP16/GOLGA7, and established roles in modifying cancer-relevant substrates such as Ras-family proteins, BiP/GRP78, CD38, STAT1, PD-L1, and PCBP1 (20–22). Therefore, ZDHHC9 should be understood as both a conserved member of the ZDHHC family and a context-specific regulator of oncogenic signaling, stress adaptation, metabolic remodeling, and tumor immune escape.

### 2.2 Structure, localization, and enzymatic properties of ZDHHC9

ZDHHC9 is a member of the zinc finger DHHC-domain-containing palmitoyl acyltransferase family, a group of integral membrane enzymes that catalyze protein S-acylation (23, 24). Like other members of this family, ZDHHC9 contains the conserved Asp-His-His-Cys (DHHC) catalytic motif that is essential for acyltransferase activity. This catalytic core enables ZDHHC9 to participate in the canonical two-step S-acylation cycle, in which the enzyme is first auto-acylated by a long-chain acyl-CoA donor and then transfers the acyl group to a cysteine residue on the substrate protein (25–27). Although palmitate is the most commonly discussed lipid donor, ZDHHC enzymes can utilize acyl chains of varying lengths, and the term S-acylation is therefore mechanistically more precise than palmitoylation in some contexts. Accordingly, in this review we use the term “palmitoylation” when referring to the most commonly studied palmitate-based modification or when describing findings reported in the original cancer studies, whereas “S-acylation” is used in a broader enzymological sense to acknowledge that ZDHHC enzymes may transfer acyl chains other than palmitate. This distinction is important because acyl-chain length and saturation can potentially influence membrane affinity, membrane microdomain partitioning, protein trafficking, and substrate residence time. However, for ZDHHC9 in cancer, most available functional studies have focused on palmitoylation-like outcomes and have not systematically compared different acyl-CoA donors. Therefore, whether ZDHHC9-dependent biological effects are differentially shaped by palmitoyl-CoA versus other acyl-CoA species remains an important unresolved question.

A distinctive feature of ZDHHC9 is that its enzymatic activity is closely linked to its interaction with the accessory protein GCP16 (also known as GOLGA7) (28). Early biochemical work established that ZDHHC9 and GCP16 form a functional complex in the Golgi apparatus and that this complex is required for robust palmitoyltransferase activity toward classical substrates such as H-Ras and N-Ras (29). Subsequent studies further showed that GCP16 does not merely serve as a passive binding partner; rather, it stabilizes ZDHHC9, promotes proper complex assembly, and helps prevent enzyme aggregation or loss of functional conformation. Thus, the active biological unit in many settings is better considered the ZDHHC9–GCP16 complex than ZDHHC9 alone.

Subcellularly, ZDHHC9 is predominantly associated with Golgi-localized membranes, which is consistent with the trafficking-dependent palmitoylation of many membrane-associated signaling proteins. This localization is biologically meaningful because S-acylation often acts as a spatial regulatory event: proteins may be palmitoylated in the Golgi and then sorted to distinct membrane compartments where their stability, signaling competence, or protein–protein interactions are further specified. In this sense, ZDHHC9 should not be viewed only as a catalytic enzyme, but also as part of a spatially organized membrane regulatory system that helps determine where and when substrate proteins acquire functional lipidation.

### 2.3 Functional consequences of protein S-palmitoylation

Protein S-palmitoylation is a reversible lipid post-translational modification in which a long-chain fatty acid, most commonly palmitate, is attached to cysteine residues through a thioester bond (30, 31). Because this bond is chemically labile, S-palmitoylation is dynamically reversible, unlike many other lipid modifications. This reversibility gives the modification switch-like properties and allows cells to rapidly tune protein behavior in response to signaling, stress, and metabolic context. The addition and removal of acyl groups are therefore part of a cyclical regulatory system rather than a static structural mark.

Functionally, S-palmitoylation can alter proteins in several non-mutually exclusive ways. First, it increases local hydrophobicity, thereby enhancing membrane affinity or stabilizing residence within specific membrane microdomains. Second, it can regulate subcellular trafficking, including Golgi-to-plasma-membrane transport, endomembrane sorting, and compartment-specific retention (32, 33). Third, it can affect protein stability, either by shielding proteins from degradation or by facilitating their assembly into more stable signaling complexes (34, 35). Fourth, it can alter protein–protein interactions, thereby influencing downstream signaling intensity, receptor function, adaptor recruitment, or scaffold formation. These properties help explain why aberrant S-palmitoylation can have broad biological consequences despite being a chemically simple lipid modification.

An important feature of S-palmitoylation biology is its close crosstalk with other post-translational regulatory systems. Palmitoylation can cooperate with or antagonize phosphorylation, ubiquitination, and intracellular degradation pathways by altering protein localization, conformational accessibility, or residence time at signaling membranes. In cancer biology, this becomes especially relevant because modest changes in membrane anchoring or protein stability can be amplified into substantial changes in signal transduction, stress tolerance, immune checkpoint expression, or metabolic adaptation. For ZDHHC9, this principle is particularly important, because several recently identified cancer-related substrates appear to be regulated not simply through membrane attachment, but through more integrated effects on protein turnover, signaling competence, and pathway output.

### 2.4 Upstream regulation and substrate selectivity of ZDHHC9

Compared with downstream functional studies, the upstream regulation of ZDHHC9 remains less fully defined. Available evidence suggests that ZDHHC9 expression may be influenced by transcriptional programs that are altered in cancer, and in at least one tumor context SP1 has been reported as an upstream transcriptional activator (36). SP1, a well-established transcription factor implicated in multiple cancers, has been reported to transcriptionally activate ZDHHC9. This regulatory axis suggests that oncogenic signals converging on SP1 could enhance ZDHHC9 expression, thereby promoting substrate-specific palmitoylation events that stabilize key proteins, amplify proliferative and survival signaling, and facilitate stress adaptation (37). Consequently, SP1-mediated upregulation of ZDHHC9 may contribute to tumor initiation, progression, and therapeutic resistance, providing a mechanistic link between transcriptional control and post-translational modulation in cancer.

However, broader regulatory mechanisms governing ZDHHC9 abundance, catalytic activation, trafficking, or substrate choice across different tumors are still insufficiently resolved. At present, it is most accurate to regard ZDHHC9 as an emerging context-responsive enzyme whose expression and functional output are likely shaped by lineage-specific transcriptional states, membrane organization, and the availability of suitable substrate proteins.

A central unresolved issue in ZDHHC9 biology is substrate selectivity. Unlike kinases, many ZDHHC enzymes do not recognize a simple linear consensus sequence. Instead, substrate engagement appears to depend on a combination of factors, including membrane colocalization, pre-existing lipid modifications, local cysteine accessibility, protein topology, and auxiliary protein interactions (38). This helps explain why ZDHHC9 can modify structurally diverse proteins in cancer-related settings, ranging from Ras-family signaling proteins to stress response molecules and immune regulators (39, 40). It also means that the biological consequences of ZDHHC9 are likely to be highly dependent on cell type and subcellular context rather than determined by a universal substrate code. Another layer of complexity arises from the reversible nature of S-acylation. The final acylation state of a given substrate reflects not only ZDHHC9-mediated acyl transfer but also the activity of depalmitoylating enzymes, including acyl-protein thioesterases (41, 42). Therefore, the functional output of ZDHHC9 should be understood as part of a broader palmitoylation–depalmitoylation equilibrium. This dynamic view is important for cancer studies, because alterations in substrate stability or signaling may arise from changes in either side of the cycle. It also has therapeutic implications: inhibiting ZDHHC9, blocking specific substrate acylation events, or shifting the balance of acylation turnover may each produce biologically distinct outcomes.

**Figure 1:**
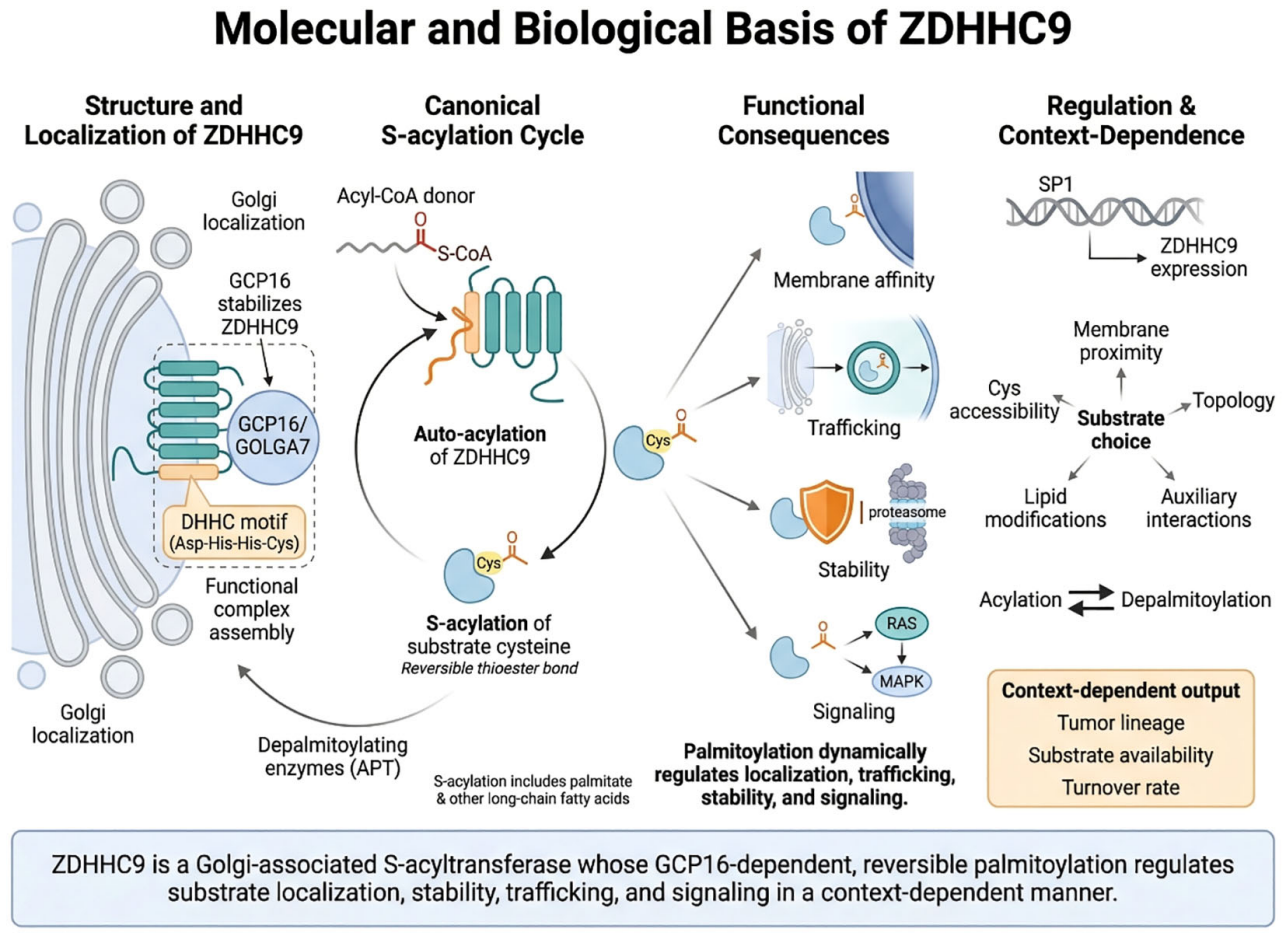
Overview of the molecular and biological basis of ZDHHC9. As a Golgi-associated DHHC family S-acyltransferase, ZDHHC9 commonly functions together with the accessory protein GCP16/GOLGA7 and mediates reversible S-acylation through a two-step catalytic process. This modification influences substrate localization, trafficking, stability, and signaling activity, whereas its overall biological output is further determined by upstream regulation, substrate selectivity, and the dynamic balance between acylation and deacylation.

Taken together, the molecular basis of ZDHHC9 points to three key concepts that are highly relevant for cancer biology. First, ZDHHC9 functions as a Golgi-associated S-acyltransferase whose activity often depends on the accessory protein GCP16. Second, through reversible S-palmitoylation, it can regulate protein localization, stability, trafficking, and signaling output. Third, its downstream effects are inherently context dependent, because both substrate availability and acylation turnover vary across tissues and tumor states. These features provide the mechanistic foundation for understanding why ZDHHC9 can influence such a wide range of malignant phenotypes in subsequent sections of this review. **Figure 1** summarizes the structural and enzymatic basis of ZDHHC9 and illustrates how reversible S-acylation controls substrate localization, stability, trafficking, and signaling. It also highlights the context-dependent nature of ZDHHC9 function, which is influenced by upstream regulation, substrate availability, and acylation turnover. In addition to acyl donor availability, the biological output of ZDHHC9 is likely to be strongly influenced by its interacting protein partners. The best-characterized partner is GCP16/GOLGA7, which stabilizes ZDHHC9 and supports formation of an active palmitoyltransferase complex (28). Beyond this canonical partner, substrate recognition may also depend on local membrane organization, Golgi retention, substrate colocalization, cysteine accessibility, and competition with other ZDHHC enzymes or depalmitoylating enzymes (4, 27, 43). Thus, ZDHHC9 function should not be interpreted as a simple enzyme–substrate reaction determined only by catalytic activity. Instead, it is better understood as a context-dependent S-acylation module in which acyl donor availability, accessory proteins, substrate repertoire, and acylation turnover collectively determine downstream effects on protein stability, trafficking, signaling, and cancer-related phenotypes.

## 3 ZDHHC9 in tumor cell-intrinsic malignant programs

Representative studies of ZDHHC9 across different cancer types are summarized in **Table 1**, highlighting its expression patterns, principal downstream pathways, biological effects, and immune relevance.

**Table 1:** Representative studies of ZDHHC9 in different cancers.

Cancer type	Expression/clinical relevance	Key substrate or pathway	Major biological effects	Immune relevance	Main implication	References
Bladder cancer	ZDHHC9 is upregulated and associated with malignant progression	BiP/GRP78 palmitoylation; UPR suppression	Promotes proliferation, inhibits apoptosis, enhances stress tolerance, increases chemoresistance	Indirect	ZDHHC9 supports proteostasis and survival adaptation in bladder cancer	(37)
Pancreatic cancer	Highly expressed and functionally important in tumor growth	CD38 palmitoylation and stabilization	Enhances tumor growth and maintains oncogenic protein stability	Indirect	Palmitoylation-dependent CD38 maintenance represents a therapeutic vulnerability	(44)
Pancreatic cancer	Associated with immune suppression and resistance to anti-PD-L1 therapy	Tumor microenvironment remodeling	Promotes immune-evasive tumor state and resistance to checkpoint blockade	Strong	Targeting ZDHHC9 may sensitize pancreatic cancer to immunotherapy	(45)
Gastric cancer	Upregulated and associated with poor prognosis	STAT1 palmitoylation-phosphorylation conversion	Promotes tumor progression and signaling activation	Potential	ZDHHC9 rewires signaling through dynamic PTM crosstalk	(46)
Gastric cancer liver metastasis	Functional role in metastatic progression	PCBP1/SLC7A11 axis; ferroptosis-related regulation	Promotes liver metastasis and suppresses ferroptosis-related cell death	Potential	ZDHHC9 contributes to metastatic fitness and redox adaptation	(47)
Lung adenocarcinoma	Promotes malignant phenotype	PD-L1 palmitoylation and stabilization	Supports tumor progression and immune escape	Strong	ZDHHC9 stabilizes checkpoint signaling at the post-translational level	(48)
Colon cancer	High ZDHHC9 correlates with poor prognosis	JAK/STAT1–PD-L1 signaling	Promotes tumor growth by suppressing effector T-cell responses	Strong	ZDHHC9 links tumor-intrinsic signaling to CD8+ T-cell dysfunction	(20)
Triple-negative breast cancer	Predicted therapeutic target and immune-associated biomarker	Immune modulation/checkpoint resistance-related programs	Associated with aggressive phenotype and potential ICB resistance	Strong	ZDHHC9 may serve as an immune-related biomarker in TNBC	(49)
Breast cancer	Prognostic and immunotherapy-related biomarker	Immune infiltration-associated signatures	Correlates with poor prognosis and immune features	Strong	ZDHHC9 may aid risk stratification and therapy prediction	(50)
Prostate cancer	Upregulated in cancer tissues	hnRNPU–SAT1 axis; spermine metabolism	Promotes proliferation and metabolic rewiring	Limited	ZDHHC9 links palmitoylation to polyamine metabolism	(21)
Osteosarcoma	Associated with Ki67 and advanced stage	KRAS-associated RAS/MAPK signaling	Promotes proliferation, migration, invasion, and survival	Limited	ZDHHC9 functions as an amplifier of oncogenic signaling	(22)

### 3.1 Promotion of tumor proliferation and survival

A recurring theme across different cancer types is that ZDHHC9 supports tumor cell proliferation and survival by stabilizing key regulatory proteins or preserving their function under stress conditions. In bladder cancer, ZDHHC9 was identified as an upregulated oncogenic factor whose depletion inhibited proliferation, increased apoptosis, and sensitized tumor cells to gemcitabine and cisplatin. Mechanistically, SP1-driven ZDHHC9 expression promoted the palmitoylation of BiP/GRP78 at Cys420, which enhanced BiP stability and preserved its endoplasmic reticulum localization, thereby suppressing unfolded protein response activation and favoring tumor cell survival. This study is important because it shows that ZDHHC9 can support malignancy not only by augmenting growth signaling, but also by buffering proteotoxic stress. A similar growth-promoting logic is seen in pancreatic cancer, where ZDHHC9-mediated palmitoylation of CD38 was shown to maintain CD38 protein abundance (44). The study demonstrated that CD38 is palmitoylated at Cys16, with ZDHHC9 functioning as the relevant palmitoyltransferase and APT1 acting as the opposing depalmitoylase. Loss of this modification reduced CD38 expression, whereas interference with CD38 palmitoylation suppressed tumor progression *in vivo*. These findings extend the role of ZDHHC9 from stress adaptation to the stabilization of a multifunctional membrane protein with tumor-promoting activity.

**Figure 2:**
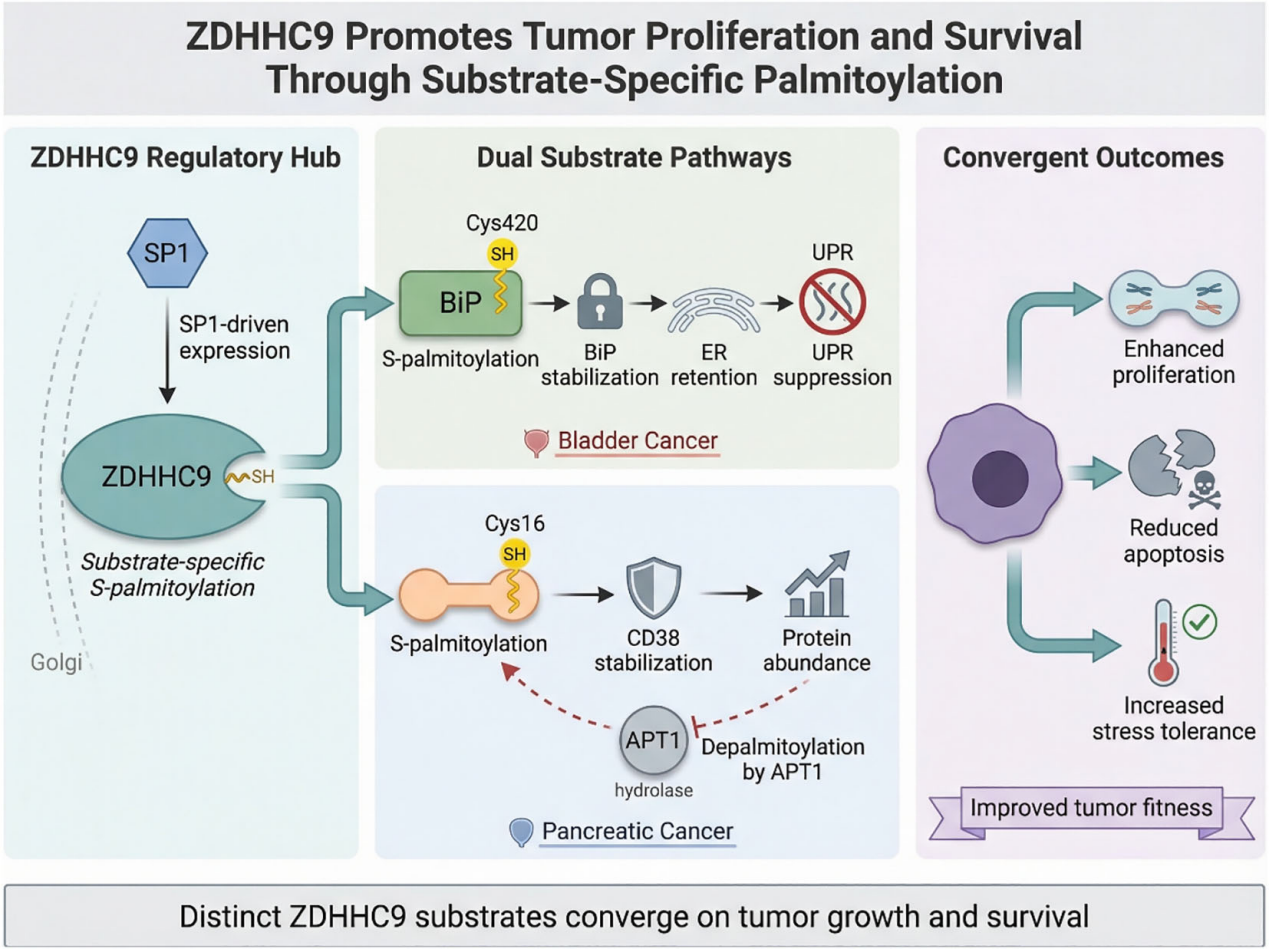
Mechanistic overview of ZDHHC9-driven tumor proliferation and survival. In bladder cancer, ZDHHC9 stabilizes BiP/GRP78 through palmitoylation, maintains its ER localization, and suppresses unfolded protein response signaling. In pancreatic cancer, ZDHHC9-mediated palmitoylation preserves CD38 abundance in opposition to APT1-dependent depalmitoylation. Together, these substrate-specific pathways converge on increased proliferation, reduced apoptosis, and enhanced tumor cell fitness.

Together, these studies support the view that ZDHHC9 enhances tumor cell fitness by maintaining the abundance or functional competence of selected substrate proteins. Rather than acting through a single universal downstream pathway, ZDHHC9 appears to promote survival through substrate-specific mechanisms that converge on a common phenotype: improved growth, reduced apoptosis, and increased tolerance to adverse conditions. **Figure 2** summarizes representative substrate-specific mechanisms by which ZDHHC9 enhances tumor proliferation and survival. Although the downstream substrates differ across tumor contexts, these palmitoylation-dependent pathways converge on improved tumor cell fitness, reduced apoptosis, and increased stress tolerance.

### 3.2 Amplification of oncogenic signaling pathways

Beyond direct effects on protein stability, ZDHHC9 also contributes to malignant progression by amplifying oncogenic signaling networks. This concept is consistent with the long-recognized connection between ZDHHC9 and Ras-family proteins, and more recent tumor studies further support its role as a signaling enhancer in cancer-relevant contexts. In osteosarcoma, ZDHHC9 expression was associated with aggressive clinicopathological features, and functional studies showed that its depletion inhibited proliferation, migration, and invasion while promoting apoptosis. Mechanistically, the authors linked ZDHHC9 to KRAS-associated activation of the RAS/MAPK pathway, supporting the interpretation that ZDHHC9 can strengthen proliferative signaling output in a tumor-promoting manner (22). An additional layer of signaling regulation is illustrated by recent work in gastric cancer. In that study, ZDHHC9 was shown to palmitoylate STAT1 and promote a palmitoylation–phosphorylation conversion process that enhanced STAT1 pathway activity and drove gastric cancer progression (46). Conceptually, this is especially notable because it moves beyond the simpler model of palmitoylation as a membrane-anchoring event and instead suggests that ZDHHC9 can influence how signaling proteins transition between biochemical states. This kind of regulatory coupling may help explain why ZDHHC9-dependent palmitoylation can produce disproportionately large downstream phenotypic effects.

**Figure 3:**
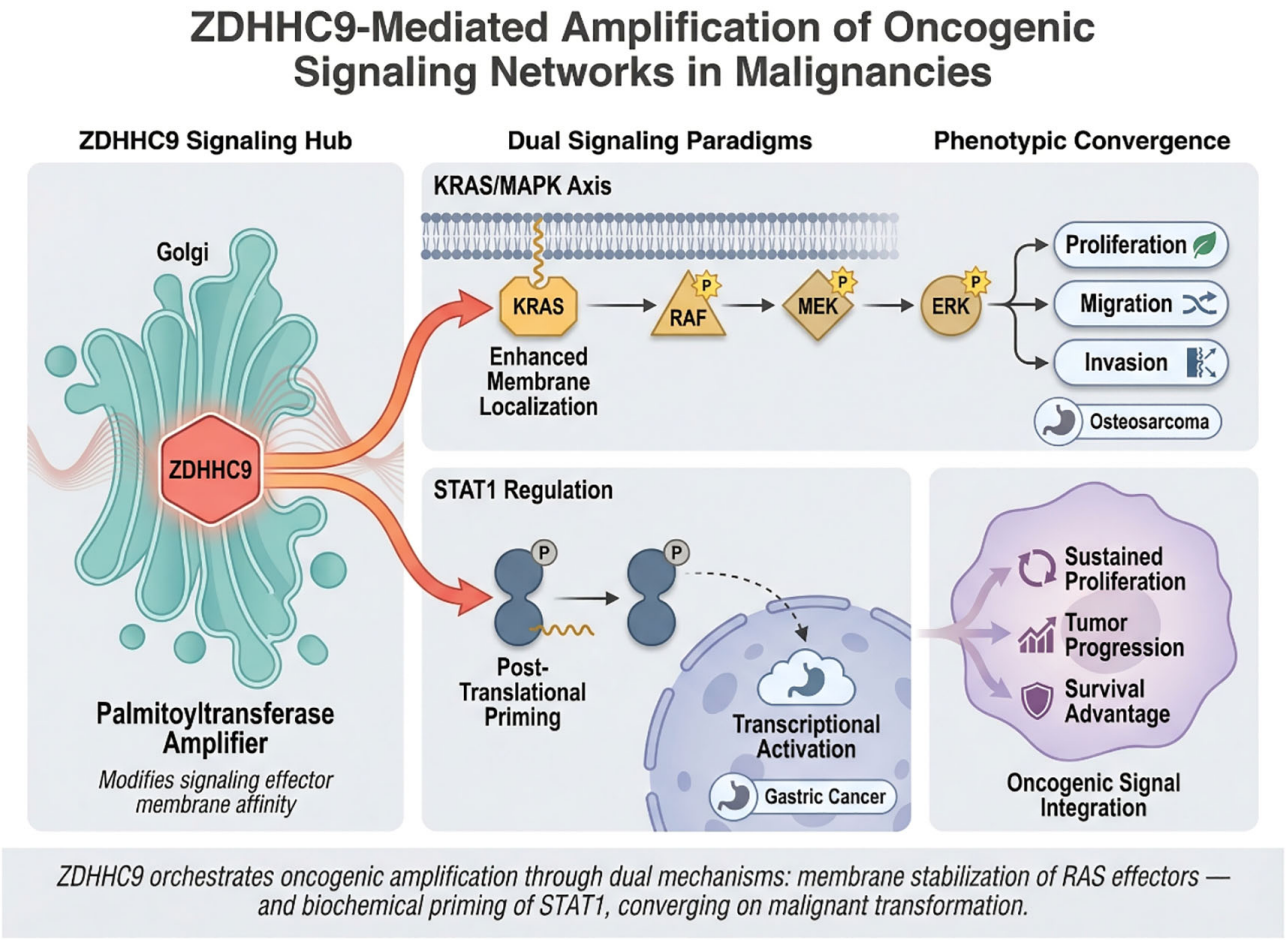
Conceptual summary of ZDHHC9-mediated amplification of oncogenic signaling pathways. In osteosarcoma, ZDHHC9 augments KRAS-associated RAS/MAPK cascade activity, thereby supporting proliferative and invasive behavior. In gastric cancer, ZDHHC9-dependent palmitoylation of STAT1 facilitates its palmitoylation–phosphorylation transition and enhances downstream signaling competence. Despite involving different signaling axes, these mechanisms converge on sustained proliferative signaling and cancer progression.

These findings support a broader model in which ZDHHC9 functions as a pathway amplifier rather than merely a passive modifying enzyme. By strengthening the stability, localization, or signaling competence of selected effectors, ZDHHC9 can reinforce the output of growth-promoting cascades such as RAS/MAPK and JAK/STAT-related networks, thereby contributing to sustained proliferative signaling in cancer cells. **Figure 3** summarizes representative mechanisms by which ZDHHC9 amplifies oncogenic signaling output in cancer. Rather than acting as a passive modifying enzyme, ZDHHC9 enhances the signaling competence of selected effectors, thereby reinforcing growth-promoting cascades such as RAS/MAPK and STAT1-related pathways.

### 3.3 Regulation of stress adaptation and proteostasis

Adaptation to cellular stress is a major requirement for tumor progression, particularly in rapidly proliferating cancers that experience proteotoxic, oxidative, and metabolic stress. Current evidence suggests that ZDHHC9 contributes to this adaptive capacity by modifying proteins involved in proteostasis and stress-response circuits. The clearest example comes from bladder cancer, where ZDHHC9-mediated palmitoylation of BiP/GRP78 suppressed unfolded protein response signaling and thereby protected tumor cells from ER stress-associated damage (37). This observation is particularly relevant because it connects ZDHHC9 to a core adaptive mechanism that allows tumor cells to survive hostile microenvironmental conditions and therapeutic challenge.

**Figure 4:**
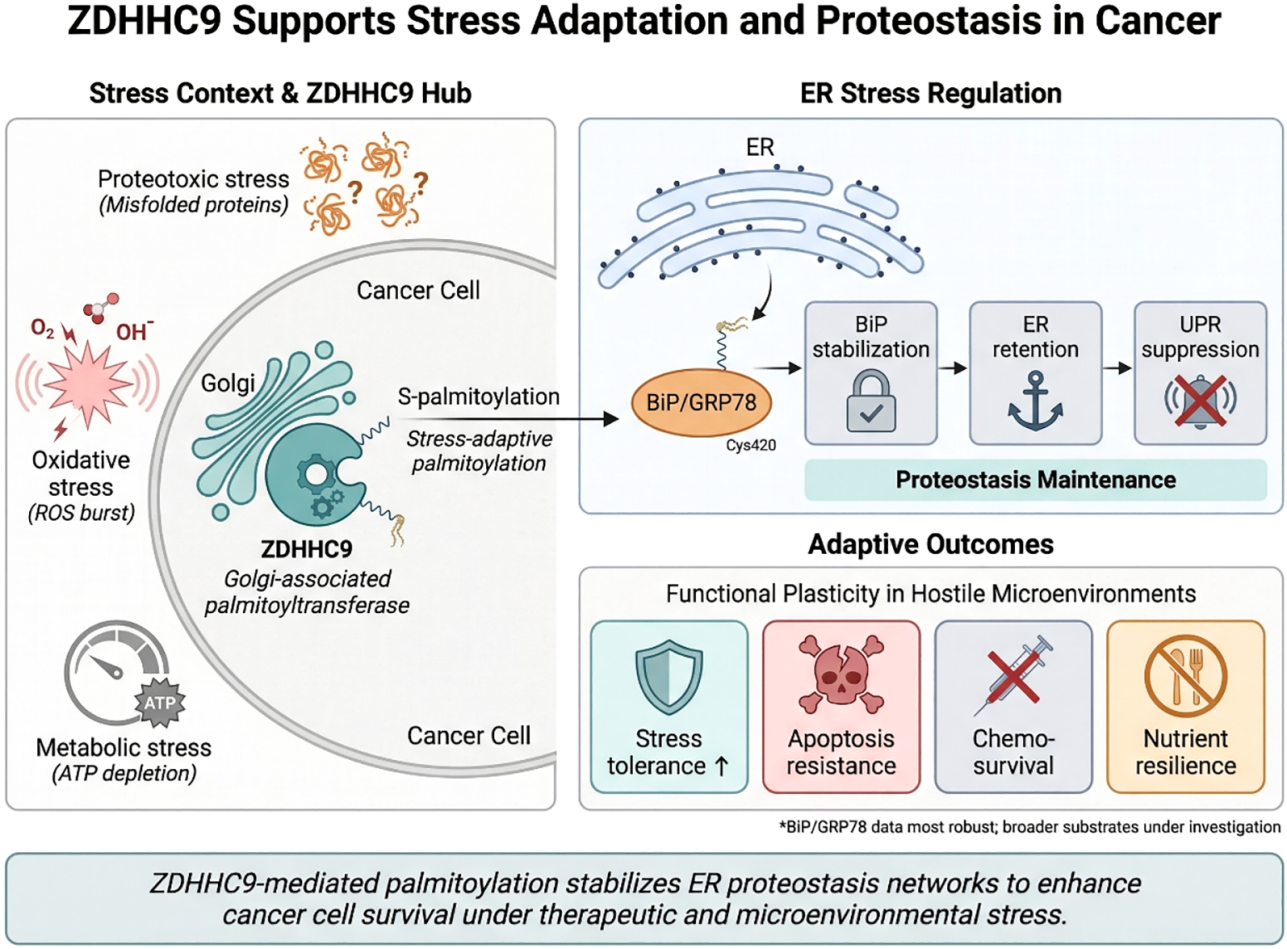
Mechanistic overview of ZDHHC9-mediated stress adaptation in cancer. By palmitoylating BiP/GRP78, ZDHHC9 enhances its stability, preserves ER localization, and suppresses unfolded protein response signaling, thereby maintaining proteostasis. This adaptive mechanism increases tumor cell tolerance to chemotherapy-associated and nutrient-restricted stress.

The implications of this biology extend beyond a single substrate. If ZDHHC9 can preserve proteostasis by stabilizing selected ER- or membrane-associated proteins, then its pro-tumor role may include a broader capacity to raise the threshold for stress-induced death. In this framework, ZDHHC9-mediated palmitoylation helps maintain a state in which cancer cells remain functionally plastic and stress tolerant, even under chemotherapy or nutrient-limited conditions. While direct evidence for this general model is still developing, available data support the idea that ZDHHC9 is involved in more than proliferative signaling alone (**Figure 4**).

### 3.4 Metabolic rewiring and ferroptosis-related pathways

Metabolic adaptation has also emerged as an important dimension of ZDHHC9 biology. In prostate cancer, ZDHHC9 was shown to promote proliferation in a palmitoylation-dependent manner while enhancing spermine catabolism (21). Mechanistically, ZDHHC9 interacted with hnRNPU and increased its stability through S-palmitoylation at Cys497 and Cys607, thereby activating the hnRNPU–SAT1 axis and linking palmitoylation to polyamine metabolism. This study is notable because it broadens the functional landscape of ZDHHC9 from signaling and protein stability to metabolic pathway control. A second metabolic and cell-death-related axis has been described in gastric cancer liver metastasis. In that model, ZDHHC9 was transcriptionally upregulated downstream of ASF1B and HOXB3 and then promoted metastasis by palmitoylating PCBP1 at C109 (47). This modification inhibited PCBP1 ubiquitination, increased its functional stability, and ultimately suppressed SLC7A11-mediated ferroptosis through effects on RNA stability. The authors further linked this axis to PD-L1 expression, suggesting that ZDHHC9 can sit at the intersection of ferroptosis regulation, metastatic fitness, and immune-related phenotypes.

**Figure 5:**
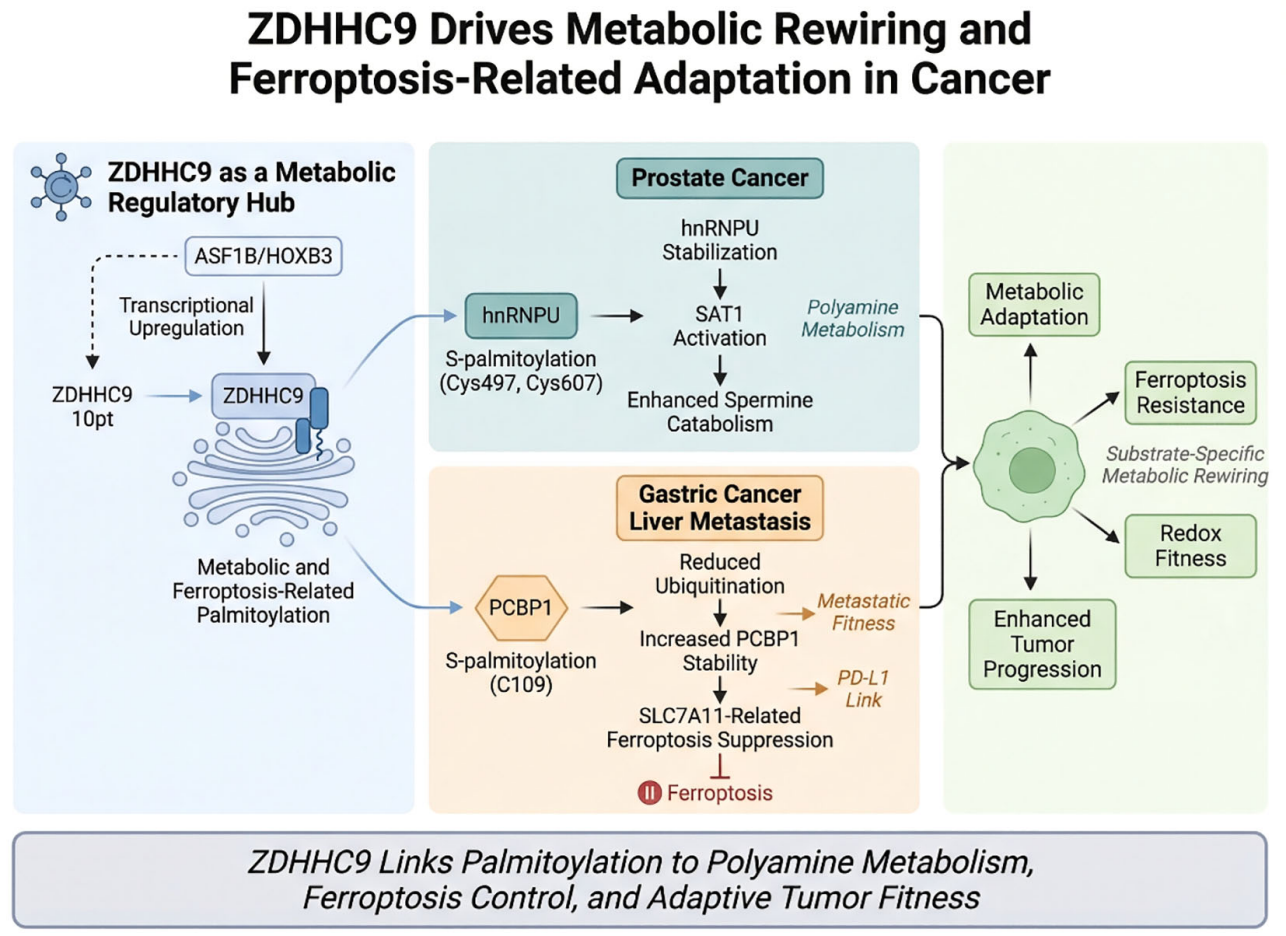
Schematic illustration of how ZDHHC9 drives metabolic rewiring and ferroptosis-related adaptation in cancer. In prostate cancer, ZDHHC9 palmitoylates hnRNPU, enhances its stability, and activates the hnRNPU–SAT1 axis to promote spermine catabolism. In gastric cancer liver metastasis, ZDHHC9-mediated palmitoylation of PCBP1 reduces its ubiquitination, increases its stability, and suppresses SLC7A11-related ferroptosis, thereby promoting metastatic fitness. These substrate-specific pathways converge on metabolic adaptation, redox fitness, and enhanced tumor progression.

Taken together, these studies indicate that ZDHHC9 can participate in metabolic rewiring through multiple substrate-specific routes. One route involves the regulation of polyamine metabolism, while another influences ferroptosis susceptibility through the PCBP1/SLC7A11 axis. This makes ZDHHC9 particularly interesting from a translational perspective, because it implies that targeting its palmitoylation program may affect not only growth signaling but also redox balance, metabolic adaptation, and cell-death sensitivity. **Figure 5** summarizes two representative substrate-specific routes by which ZDHHC9 contributes to metabolic rewiring in cancer. Through regulation of polyamine metabolism and ferroptosis-related pathways, ZDHHC9 extends its oncogenic role beyond signaling amplification to the control of redox adaptation, cell-death sensitivity, and metastatic fitness.

### 3.5 Context-dependent functions and biological heterogeneity

Although most available studies support a tumor-promoting role for ZDHHC9, the literature also argues against an overly simplified interpretation. The downstream effects of ZDHHC9 vary substantially depending on tumor type, substrate repertoire, and cellular context. In some models, the dominant phenotype is proliferative signaling; in others, it is immune suppression, proteostasis control, or ferroptosis resistance (37, 44, 49). This variability likely reflects the fact that ZDHHC9 does not impose a single program on all tumors, but instead modifies whichever substrates are available and functionally relevant in a given biological setting. This context dependence is important for both interpretation and therapeutic development. It suggests that ZDHHC9 should not be described as a universal master regulator across all cancers. A more balanced conclusion is that ZDHHC9 acts as an emerging, context-responsive palmitoyltransferase whose tumor-promoting effects are frequently strong but mechanistically heterogeneous. This view better fits the current evidence and provides a more rigorous conceptual bridge to the next section, where its role in tumor immune regulation becomes especially prominent. The major substrates and mechanistic consequences of ZDHHC9-mediated palmitoylation identified to date are summarized in **Table 2**, underscoring how substrate-specific regulation converges on tumor growth, stress adaptation, metabolic rewiring, and immune evasion.

**Table 2:** Major substrates and mechanistic consequences of ZDHHC9-mediated palmitoylation in cancer.

Substrate/axis	Cancer context	Effect of ZDHHC9-mediated palmitoylation	Downstream consequence	Biological outcome	References
BiP/GRP78	Bladder cancer	Increases protein stability and preserves ER localization	Suppresses unfolded protein response (UPR) activation	Enhanced survival, stress adaptation, and chemotherapy tolerance	(37)
CD38	Pancreatic cancer	Stabilizes CD38 protein expression	Sustains tumor-promoting signaling and cellular fitness	Accelerated pancreatic cancer growth	(44)
STAT1	Gastric cancer	Promotes palmitoylation-phosphorylation conversion	Enhances STAT1 signaling output	Increased gastric cancer progression	(46)
PD-L1	Lung adenocarcinoma	Increases PD-L1 stability by palmitoylation	Reduces PD-L1 degradation and enhances immune checkpoint signaling	Immune escape and tumor progression	(48)
JAK/STAT1–PD-L1 axis	Colon cancer	Enhances IFN-γ-induced signaling and PD-L1 expression	Suppresses CD8+ T-cell cytotoxicity	Tumor immune evasion	(20)
PCBP1	Gastric cancer liver metastasis	Inhibits ubiquitination and increases functional stability	Regulates SLC7A11-related ferroptosis resistance	Increased metastasis and reduced ferroptotic vulnerability	(47)
hnRNPU	Prostate cancer	Stabilizes hnRNPU through S-palmitoylation	Activates SAT1 and spermine catabolism	Metabolic rewiring and tumor growth	(21)
Tumor immune microenvironment	Pancreatic cancer	Remodels tumor state toward immune suppression	Restrains CD8+ T-cell-dependent anti-tumor response	Resistance to anti-PD-L1 therapy	(45)

## 4 ZDHHC9 and tumor immune regulation

### 4.1 Shaping an immunosuppressive tumor microenvironment

**Figure 6:**
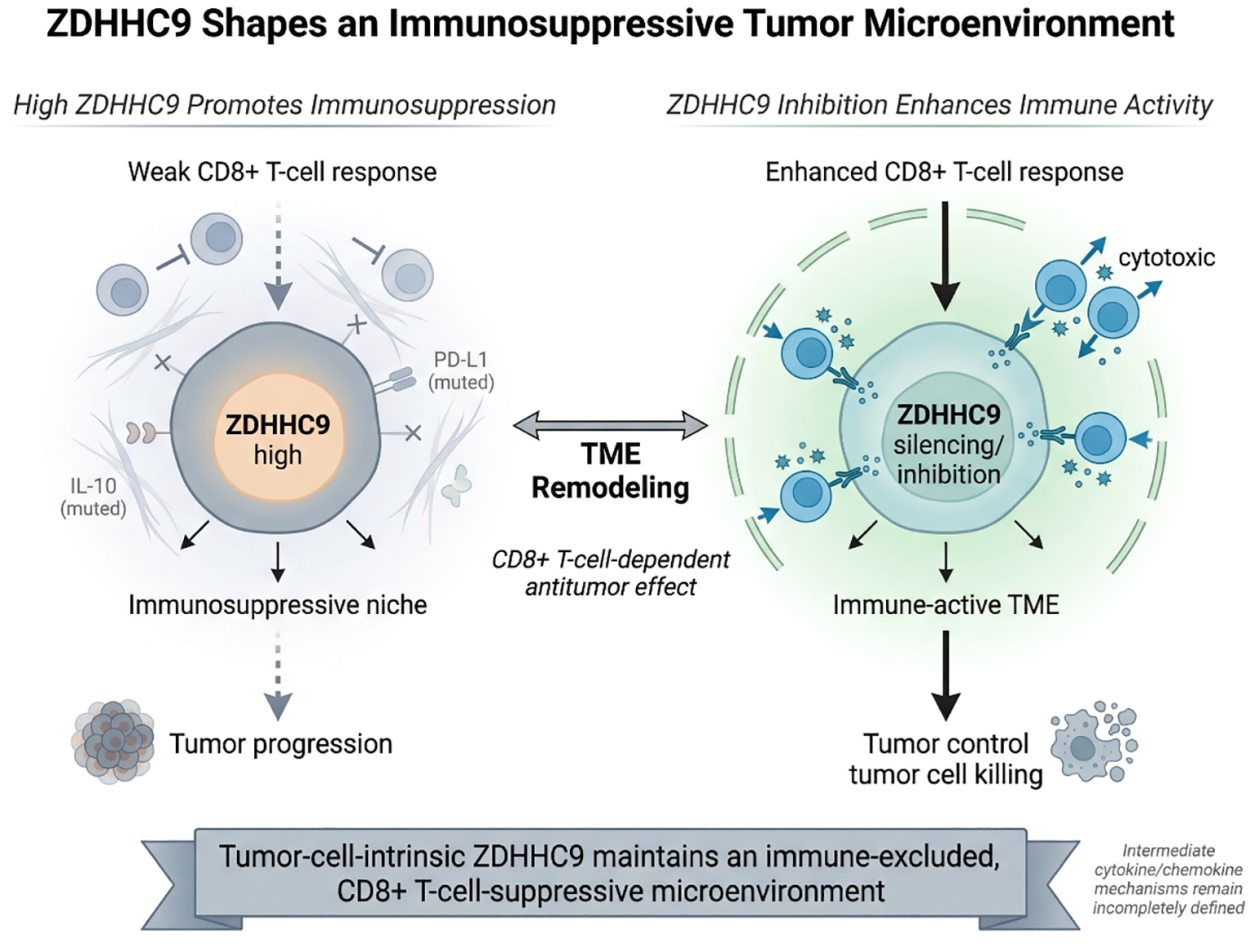
Schematic illustration of how ZDHHC9 shapes an immunosuppressive tumor microenvironment. In pancreatic cancer, high tumor-cell-intrinsic ZDHHC9 is associated with maintenance of an immune-suppressive niche characterized by reduced CD8+ T-cell activity and enhanced tumor growth. By contrast, ZDHHC9 inhibition remodels the microenvironment toward a more immune-active state, enhances CD8+ T-cell-dependent antitumor responses, and suppresses tumor progression.

Recent studies suggest that the biological impact of ZDHHC9 extends beyond tumor cell-intrinsic proliferation and survival and includes a significant role in shaping the tumor immune microenvironment (45, 50). The strongest functional evidence comes from pancreatic cancer, where ZDHHC9 overexpression was linked to suppression of host anti-tumor immunity. In that model, silencing ZDHHC9 reduced tumor growth in immunocompetent mice, prolonged survival, and remodeled the tumor microenvironment toward a more immune-active state, indicating that ZDHHC9 contributes to the maintenance of an immunosuppressive niche. Importantly, the anti-tumor effect of ZDHHC9 inhibition was largely dependent on CD8+ T cells, supporting the idea that ZDHHC9 influences tumor progression partly by restricting effective cytotoxic immunity (51, 52). This immunomodulatory role is conceptually important because it places ZDHHC9 within the broader framework of tumor cell-intrinsic determinants of immune exclusion. Rather than functioning solely as a metabolic or signaling enzyme, ZDHHC9 appears capable of altering how tumor cells interact with their immune surroundings. The available evidence does not yet define all intermediary cytokine or chemokine pathways responsible for this effect, but current data support the interpretation that ZDHHC9 activity helps preserve a microenvironment less permissive to productive anti-tumor T-cell responses (**Figure 6**).

### 4.2 Inhibition of effector T-cell responses

**Figure 7:**
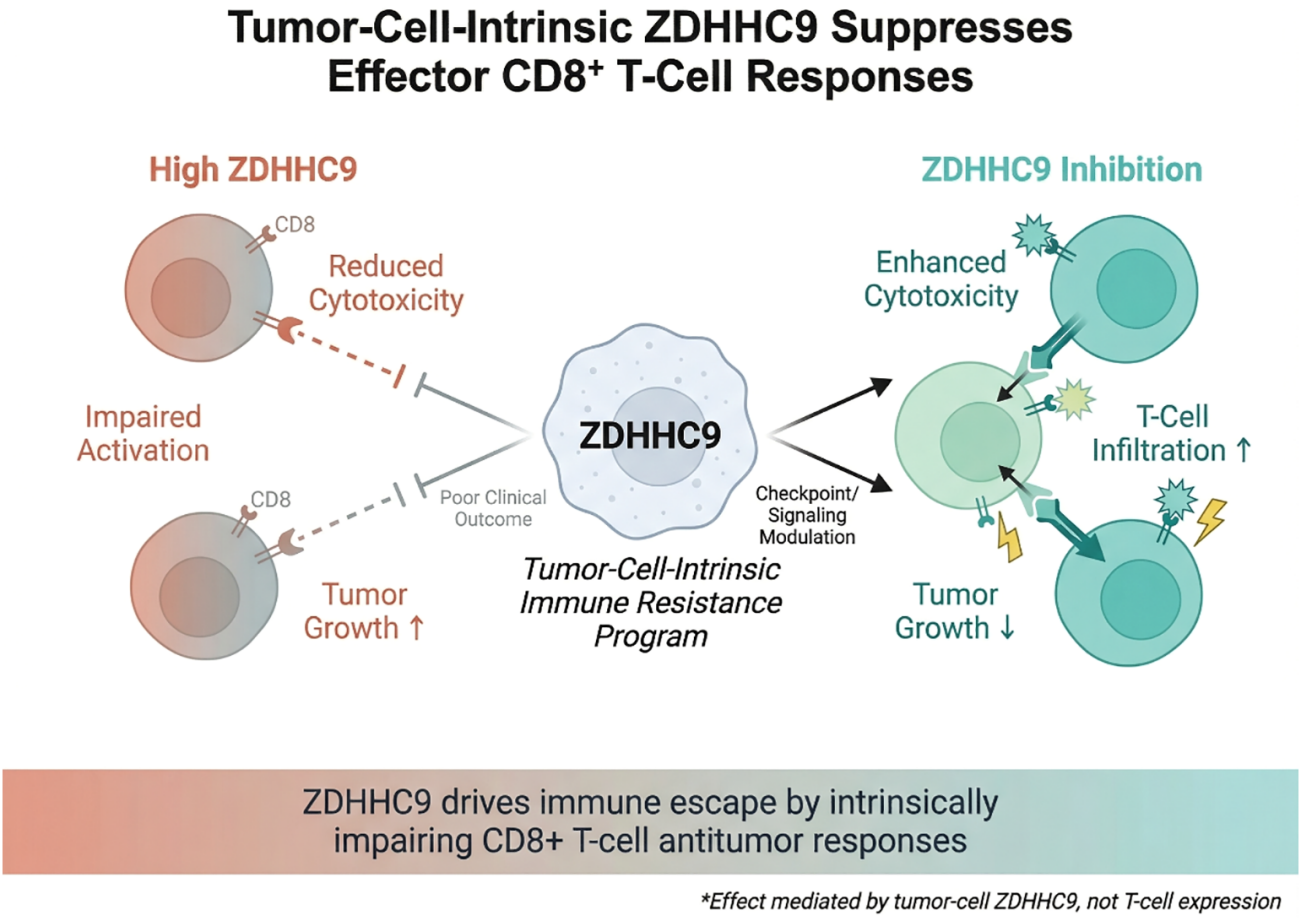
Schematic illustration of how tumor-cell-intrinsic ZDHHC9 suppresses effector CD8+ T-cell responses. In colon cancer, high ZDHHC9 expression in tumor cells is associated with reduced CD8+ T-cell cytotoxicity and limited antitumor killing, contributing to tumor growth. By contrast, ZDHHC9 inhibition enhances CD8+ T-cell infiltration, activation, and cytotoxic function, thereby improving tumor control.

Evidence from colon cancer provides a more direct link between ZDHHC9 and effector T-cell suppression (20). In that study, ZDHHC9 was highly expressed in colon cancer and associated with worse prognosis. Functionally, ZDHHC9 knockdown reduced tumor growth *in vivo* despite not producing a simple anti-proliferative effect *in vitro*, suggesting that immune-dependent mechanisms were central to the phenotype. The authors showed that inhibition of ZDHHC9 enhanced CD8+ T-cell-mediated cytotoxicity *in vitro* and increased CD8+ T-cell infiltration and activation in tumors, indicating that ZDHHC9-expressing cancer cells can restrain effector T-cell function (53, 54). These findings are especially relevant for a mechanistic review because they illustrate how a palmitoyltransferase can influence anti-tumor immunity indirectly through tumor-cell signaling states. In other words, ZDHHC9 does not need to be expressed in immune cells themselves to exert immunological effects; by altering signaling and checkpoint-related programs within cancer cells, it can create conditions that weaken T-cell killing. This makes ZDHHC9 a useful example of how post-translational lipidation machinery can contribute to immune escape as part of a tumor cell-intrinsic immune resistance program. **Figure 7** highlights the concept that ZDHHC9 can suppress antitumor immunity through tumor-cell-intrinsic mechanisms. By altering the signaling state of cancer cells, ZDHHC9 creates conditions that weaken CD8+ T-cell-mediated killing, whereas its inhibition restores effector T-cell activity and reduces tumor growth.

### 4.3 Regulation of immune checkpoint signaling

**Figure 8:**
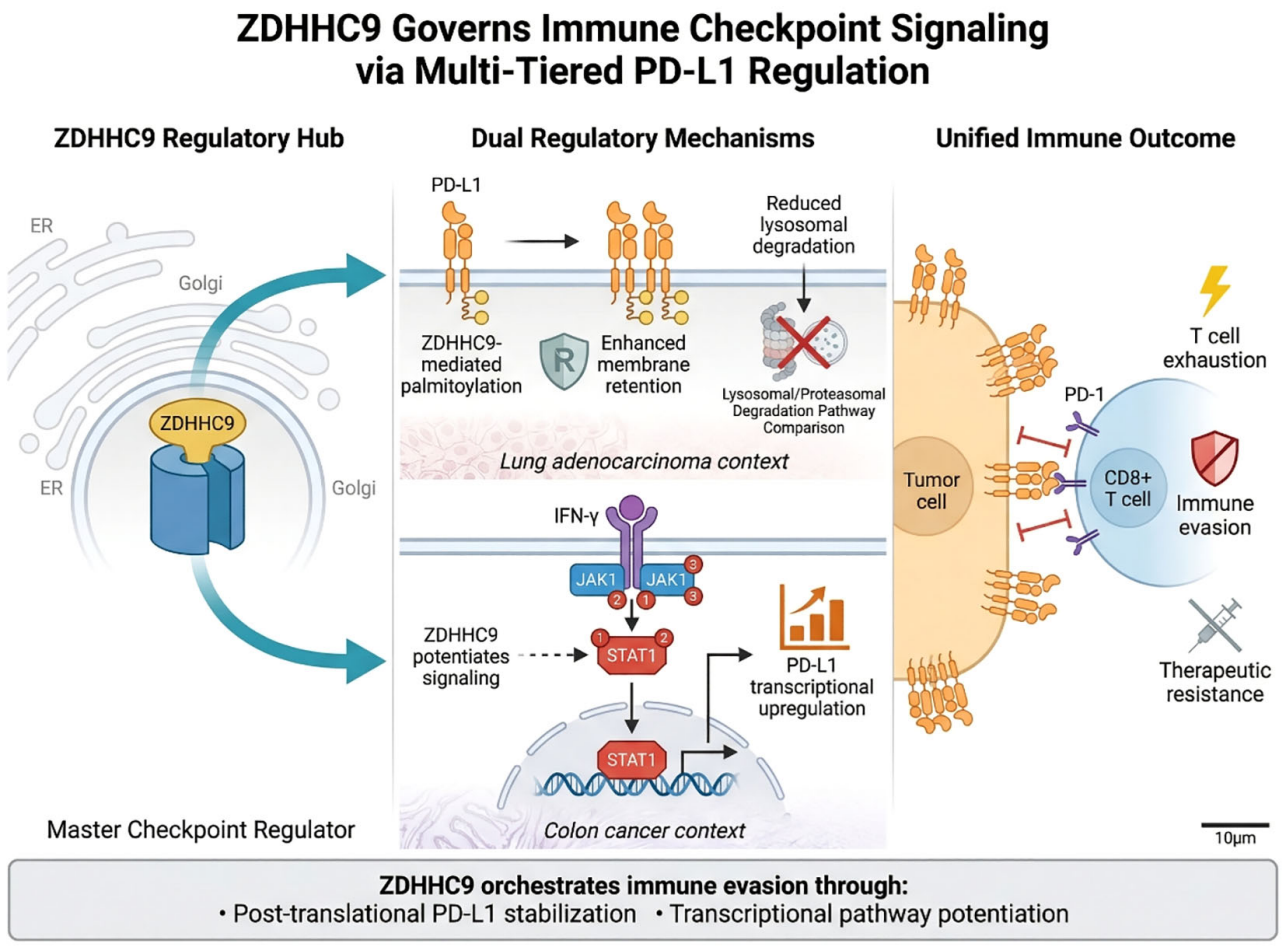
Conceptual summary of ZDHHC9-mediated checkpoint regulation in cancer. In lung adenocarcinoma, ZDHHC9 supports immune checkpoint competence by directly palmitoylating PD-L1 and protecting it from degradation. In colon cancer, ZDHHC9 indirectly promotes PD-L1 expression by reinforcing IFN-γ–JAK1/STAT1 signaling. Despite acting through different mechanisms, both pathways converge on impaired CD8+ T-cell activity, immune escape, and malignant progression.

One of the most consistent immune-related themes in the current ZDHHC9 literature is its connection to PD-L1 regulation. In lung adenocarcinoma, ZDHHC9 was shown to promote PD-L1 palmitoylation and thereby increase PD-L1 protein stability (48). ZDHHC9 knockdown reduced PD-L1 palmitoylation, accelerated PD-L1 degradation, and suppressed malignant phenotypes, supporting a model in which ZDHHC9 sustains immune evasion by protecting PD-L1 from turnover (55). This study is important because it links ZDHHC9 to immune checkpoint biology at the level of post-translational control rather than only transcriptional activation. In colon cancer, the link between ZDHHC9 and PD-L1 was connected to IFN-γ-induced JAK/STAT1 signaling. There, ZDHHC9 increased JAK1 and STAT1 phosphorylation and upregulated PD-L1 expression, which in turn impaired CD8+ T-cell function. Thus, across different tumor settings, ZDHHC9 appears able to support checkpoint signaling through more than one mechanism: by stabilizing PD-L1 protein directly in one context and by enhancing upstream signaling that promotes PD-L1 expression in another. Taken together, these findings suggest that ZDHHC9 may serve as a multi-level regulator of checkpoint competence in cancer cells (**Figure 8**).

### 4.4 Implications for immunotherapy response

The emerging relationship between ZDHHC9 and immune suppression naturally raises the question of whether ZDHHC9 influences responsiveness to immunotherapy. The clearest preclinical evidence again comes from pancreatic cancer, where inactivation of ZDHHC9 enhanced the anti-tumor efficacy of anti-PD-L1 therapy (45). In that model, targeting ZDHHC9 not only inhibited tumor growth on its own but also acted as an immunotherapeutic booster, indicating that ZDHHC9 activity may contribute to primary resistance to checkpoint blockade. These findings support the idea that ZDHHC9 is not merely correlated with immune dysfunction, but may be functionally involved in determining whether tumors respond to immune checkpoint inhibition.

Additional support for this concept comes from breast cancer studies, particularly in triple-negative breast cancer, where bioinformatic and translational analyses identified ZDHHC9 as a candidate biomarker associated with immune modulation and immune checkpoint blockade resistance (49, 50). A later systematic analysis in breast cancer likewise suggested that ZDHHC9 expression correlates with immune features and may have value as a prognostic and immunotherapy-related biomarker. Although these breast cancer studies are more associative than mechanistically definitive, they reinforce the broader view that ZDHHC9 may influence the immune landscape in ways relevant to patient stratification and treatment response.

### 4.5 Current interpretation and remaining gaps

Taken together, current evidence supports a model in which ZDHHC9 promotes tumor immune evasion through at least three interconnected routes: by fostering an immunosuppressive tumor microenvironment, by weakening effector CD8+ T-cell responses, and by strengthening immune checkpoint signaling, especially PD-L1-related programs. These observations make ZDHHC9 particularly notable among palmitoyltransferases, because its effects are not restricted to tumor growth control but extend to immunological determinants of therapy response. At the same time, the field is still early. Most mechanistic data come from a limited number of tumor models, and the precise substrate spectrum and context-specific determinants of ZDHHC9-mediated immune regulation remain incompletely defined. A balanced interpretation, therefore, is that ZDHHC9 is an emerging but not yet fully resolved regulator of tumor immunity whose translational relevance appears promising and warrants deeper investigation.

## 5 Therapeutic targeting of ZDHHC9 in cancer

### 5.1 Rationale for therapeutic intervention

The growing interest in ZDHHC9 as a therapeutic target stems from the fact that its biological effects span several cancer-relevant layers at once. As summarized above, ZDHHC9 can enhance tumor cell proliferation and survival, reinforce stress adaptation, reshape metabolic programs, and promote immune evasion through checkpoint-related pathways (56, 57). In practical terms, this means that ZDHHC9 sits at a strategically attractive position: inhibiting it could, at least in principle, weaken malignant fitness while simultaneously improving anti-tumor immunity. This dual relevance is particularly important in tumors where conventional cytotoxic therapy alone is insufficient and where immune checkpoint blockade faces intrinsic resistance. Another reason ZDHHC9 is therapeutically interesting is that it operates at the level of reversible post-translational modification. Unlike fixed genetic lesions, palmitoylation-dependent signaling is dynamic and potentially druggable. Because S-acylation controls protein stability, trafficking, and pathway output, interference with a key palmitoyltransferase such as ZDHHC9 may disrupt several downstream phenotypes without requiring direct inhibition of every substrate individually. At the same time, this promise must be interpreted cautiously, because the same feature that makes ZDHHC9 broadly influential also raises concerns about pleiotropy, tissue specificity, and context-dependent response.

### 5.2 Direct inhibition or silencing of ZDHHC9

The most straightforward therapeutic concept is to reduce ZDHHC9 activity or expression directly. Preclinical support for this approach is strongest in pancreatic cancer, where genetic depletion of ZDHHC9 suppressed tumor progression, prolonged survival in mouse models, and enhanced the efficacy of anti-PD-L1 therapy. Notably, that study also used a ZDHHC9-siRNA nanoparticle system to silence ZDHHC9 in tumors, providing proof of concept that tumor-directed ZDHHC9 suppression is feasible beyond standard *in vitro* knockdown experiments (45, 58–61). This is an important translational step, because it moves the field from descriptive mechanism toward actionable intervention. However, true small-molecule selective inhibitors of ZDHHC9 are not yet established as standard tools in the way kinase inhibitors are. A major challenge across the DHHC family is that the catalytic architecture is highly conserved, making isoform-selective inhibition difficult. In addition, many studies in the broader palmitoylation field have relied on 2-bromopalmitate (2-BP), which is widely recognized as a non-selective inhibitor of lipid metabolism and protein palmitoylation rather than a precise ZDHHC9-directed agent (62, 63). As a result, although broad palmitoylation inhibition can be experimentally informative, it does not solve the specific translational problem of how to inhibit ZDHHC9 with sufficient selectivity and acceptable toxicity.

### 5.3 Targeting ZDHHC9-dependent substrate palmitoylation

An alternative strategy is not to inhibit ZDHHC9 globally, but instead to block critical substrate-specific palmitoylation events. This concept has already gained experimental support in pancreatic cancer. In the CD38 study, ZDHHC9-mediated palmitoylation stabilized CD38, and the authors designed a competitive peptide to interfere with this modification (44). Blocking CD38 palmitoylation reduced CD38 expression and inhibited tumor progression *in vivo*, illustrating that one can therapeutically exploit a ZDHHC9-controlled substrate without necessarily needing a fully selective enzyme inhibitor (64, 65). This substrate-centered approach may be especially attractive for ZDHHC9 because its downstream phenotypes are mechanistically heterogeneous. In one tumor, the most actionable output may be PD-L1 stabilization; in another, it may be BiP/GRP78-dependent stress buffering, CD38 maintenance, or STAT1 signaling conversion. A substrate-specific strategy could therefore improve therapeutic precision by focusing on the disease-relevant palmitoylation event rather than suppressing all ZDHHC9 functions indiscriminately. The drawback, however, is that such an approach requires detailed structural and mechanistic knowledge of each substrate interface, which is still incomplete for much of the ZDHHC9 network.

### 5.4 Combination therapy opportunities

Current evidence suggests that ZDHHC9-targeted intervention may be especially valuable in combination therapy rather than as a stand-alone strategy. The clearest case is immunotherapy: in pancreatic cancer, ZDHHC9 deficiency sensitized tumors to anti-PD-L1 treatment in a CD8+ T-cell-dependent manner, supporting the idea that ZDHHC9 inhibition can function as an immunotherapeutic booster. This is conceptually important because pancreatic cancer is well known to respond poorly to immune checkpoint blockade alone, largely because of its profoundly immunosuppressive microenvironment. Thus, targeting ZDHHC9 may help convert checkpoint-resistant tumors into a more immunologically permissive state.

Combination strategies may also be relevant for chemotherapy sensitization. In bladder cancer, ZDHHC9 knockdown enhanced the efficacy of gemcitabine and cisplatin while promoting apoptosis, suggesting that ZDHHC9 contributes to treatment tolerance at least in part through stress adaptation and BiP/GRP78-dependent UPR suppression (66–68). These findings raise the possibility that ZDHHC9 inhibition could lower the survival threshold of tumor cells exposed to cytotoxic stress, thereby improving responses to standard chemotherapy. A broader implication is that ZDHHC9-directed strategies could be paired with therapies aimed at ferroptosis, metabolic vulnerability, or stress-response pathways in tumors where these outputs dominate. Although this concept remains more inferential than clinically validated, it is grounded in recent studies linking ZDHHC9 to polyamine metabolism, ferroptosis-related control, and adaptive signaling programs. In this sense, ZDHHC9 may be best viewed as a combinatorial target whose therapeutic value depends on identifying the dominant downstream dependency in each tumor context.

### 5.5 Challenges in clinical translation

Despite the promise of this field, several barriers currently limit clinical translation. First, the substrate spectrum of ZDHHC9 is still incompletely defined across tumor types, and recent technology papers emphasize that mapping the proteome-wide targets of a specific ZDHHC remains technically challenging, though increasingly feasible. Without a fuller substrate map, it is difficult to predict which tumors will respond best to ZDHHC9 inhibition or which toxicities may emerge in normal tissues. Second, ZDHHC9 biology is context dependent. Most available studies support a tumor-promoting role, but the dominant output varies across cancers, ranging from immune suppression to stress adaptation to metabolic rewiring. This heterogeneity complicates biomarker development and implies that a one-size-fits-all ZDHHC9-targeting strategy is unlikely to be optimal. Patient selection will probably require integration of ZDHHC9 expression, substrate status, immune phenotype, and perhaps tumor lineage-specific signaling dependencies. Third, the field still lacks mature pharmacological tools. Broad palmitoylation inhibitors such as 2-BP are useful experimental probes but suffer from poor selectivity, and the highly conserved catalytic features of DHHC enzymes make isoform-specific drug development difficult (15). For that reason, future progress may depend on several parallel advances: better structural understanding of ZDHHC9, better substrate-mapping technologies, rational design of interface-disrupting molecules, and delivery platforms that enable tumor-restricted silencing or degradation. Until such tools are available, the therapeutic potential of ZDHHC9 remains compelling but still predominantly preclinical.

### 5.6 Overall perspective

Taken together, current evidence supports ZDHHC9 as a promising but still early-stage therapeutic target in oncology. The most credible translational scenarios at present are those in which ZDHHC9 inhibition is used to sensitize tumors to immunotherapy, enhance chemotherapy response, or disrupt a specific palmitoylation-dependent oncogenic substrate. What distinguishes ZDHHC9 from many other cancer-related enzymes is not simply that it promotes tumor growth, but that it connects malignant signaling with immune and stress-adaptive programs. That integrative position makes it attractive, but also means that therapeutic development must proceed with mechanistic precision rather than broad generalization. Current therapeutic strategies and translational opportunities for targeting ZDHHC9 are summarized in **Table 3**, including direct inhibition, substrate-specific interference, and combination approaches with chemotherapy or immunotherapy.

**Table 3:** Therapeutic implications and future translational directions of targeting ZDHHC9.

Therapeutic strategy	Mechanistic rationale	Current evidence	Potential advantages	Major limitations/challenges	References
Direct inhibition of ZDHHC9 enzyme activity	Block palmitoylation of multiple oncogenic substrates simultaneously	Strong preclinical rationale, but limited selective inhibitors	Broad anti-tumor impact across signaling, metabolism, and immunity	Lack of isoform-selective inhibitors; possible off-target effects	(44, 45)
Genetic silencing of ZDHHC9 (siRNA/shRNA/nanoparticle)	Reduce ZDHHC9 expression in tumors	Demonstrated in pancreatic cancer models	Proof of concept for tumor-directed intervention; may enhance immunotherapy	Delivery efficiency, tissue specificity, and clinical feasibility remain challenging	(20, 45)
Substrate-specific blockade of palmitoylation	Disrupt critical downstream dependency without global enzyme inhibition	Competitive peptide strategy shown for CD38	More precise and potentially less toxic than pan-ZDHHC9 inhibition	Requires detailed structural knowledge of each substrate interface	(44)
Combination with immune checkpoint blockade	Reverse immune suppression and improve T-cell activity	Supported by pancreatic cancer and immune-related studies	Particularly attractive for immune-cold or checkpoint-resistant tumors	Tumor-type specificity and predictive biomarkers still unclear	(45, 48)
Combination with chemotherapy	Lower stress-adaptive survival threshold and sensitize tumor cells	Supported by bladder cancer data with gemcitabine/cisplatin	Could improve efficacy of standard treatments	May depend on dominant downstream substrate and tumor context	(37)
Combination with ferroptosis- or metabolism-targeted therapy	Exploit ZDHHC9-linked metabolic rewiring and ferroptosis control	Conceptually supported by gastric cancer and prostate cancer studies	Expands therapeutic scope beyond proliferation control	Still largely preclinical and mechanistically incomplete	(21, 47)
Biomarker development	Use ZDHHC9 to stratify prognosis, immune state, or treatment benefit	Supported by breast cancer and TNBC analyses	May guide patient selection for immunotherapy or targeted combinations	Expression alone may be insufficient; requires integrated biomarker models	(49, 50)
Future precision strategies	Target the dominant ZDHHC9-dependent substrate network in each tumor	Emerging direction	Higher mechanistic precision and better translational potential	Requires substrate mapping, spatial profiling, and better pharmacological tools	(44, 45)

## 6 Outstanding questions and future perspectives

Despite the rapid expansion of studies linking ZDHHC9 to tumor progression and immune regulation, the field remains at an early mechanistic stage. Current evidence clearly supports the idea that ZDHHC9 is more than a housekeeping palmitoyltransferase, yet several fundamental questions remain unresolved. Addressing these issues will be essential for moving from descriptive associations and isolated substrate studies toward a coherent and clinically actionable framework. The next phase of research will likely depend not only on additional tumor models, but also on better substrate-mapping technologies, more refined spatial analyses, and more precise targeting strategies. Whole-proteome methods to map ZDHHC-specific S-acylation have recently become feasible, offering a path to define enzyme-specific substrate networks much more systematically than was previously possible.

### 6.1 Defining the substrate landscape of ZDHHC9 across tumor contexts

One of the most important unresolved questions is which proteins are genuinely and recurrently regulated by ZDHHC9 in different cancers. Current knowledge is still derived largely from tumor-specific studies that each identify a limited set of functionally validated substrates, such as BiP/GRP78, CD38, STAT1, PD-L1, and PCBP1. While these studies are highly informative, they do not yet reveal whether there is a shared “core” ZDHHC9 substrate program across cancers or whether most ZDHHC9 outputs are lineage-specific. Because recent proteomic technology can now map ZDHHC-specific S-acylation at scale, future studies should use these platforms to compare substrate repertoires across tumor types, treatment conditions, and immune states. Such work would help distinguish universal ZDHHC9 functions from highly context-restricted ones and would substantially improve both mechanistic clarity and therapeutic prioritization.

### 6.2 Understanding how ZDHHC9 selects substrates and integrates with other post-translational modifications

A related challenge is to explain how ZDHHC9 selects one substrate over another in a given biological setting. Unlike kinases, ZDHHC enzymes do not appear to operate through a simple linear recognition motif alone, and substrate engagement likely depends on membrane colocalization, cysteine accessibility, local topology, accessory factors, and competition with other acyltransferases or depalmitoylases (69, 70). This complexity may be one reason why ZDHHC9 can support distinct phenotypes in different cancers, ranging from checkpoint regulation to stress adaptation to metabolic rewiring. The field also needs a more systematic understanding of how ZDHHC9-mediated palmitoylation intersects with phosphorylation, ubiquitination, and degradation pathways. Individual studies already point in this direction—for example, ZDHHC9-linked regulation of STAT1 signaling and of PCBP1 ubiquitination—but these examples have not yet been integrated into a broader mechanistic model. Future work should therefore move beyond the question of “what is palmitoylated” to ask “how palmitoylation reshapes PTM crosstalk and pathway state transitions.”

### 6.3 Clarifying the immune ecosystem effects of ZDHHC9

The evidence connecting ZDHHC9 to tumor immunity is promising, but much remains unresolved. At present, the most convincing studies show that ZDHHC9 can suppress anti-tumor immunity, increase PD-L1-related immune escape, and reduce responsiveness to anti-PD-L1 therapy in selected models (71–73). However, it is still unclear whether these effects are driven primarily by tumor-cell-intrinsic checkpoint biology, by changes in cytokine and chemokine networks, by altered metabolic competition in the tumor microenvironment, or by some combination of all three. This distinction matters because each mechanism would imply different biomarkers and different combination strategies. Future studies should therefore incorporate immune-competent models, perturbation experiments in defined stromal and immune compartments, and spatial profiling approaches capable of resolving how ZDHHC9 expression relates to CD8+ T-cell exclusion, macrophage polarization, or tertiary lymphoid organization (74–76). Current breast cancer analyses suggesting that ZDHHC9 may act as an immunotherapy-related biomarker further reinforce the need for more mechanistically grounded immune stratification studies.

### 6.4 Moving from bulk expression to spatial and single-cell resolution

Most current ZDHHC9 studies still rely on bulk tumor analyses or conventional cell-line systems. While these approaches have been useful for establishing proof of principle, they are limited in their ability to capture the full heterogeneity of tumor ecosystems. Because palmitoylation-dependent signaling is highly context sensitive, future progress will likely require integrating single-cell transcriptomics, spatial transcriptomics, and spatial proteomics with functional acylation assays. Such approaches could identify which tumor subpopulations express high ZDHHC9, whether ZDHHC9-high regions colocalize with immune exclusion or stress-adapted niches, and whether specific substrate programs dominate in metastatic versus primary lesions. In a field where biological interpretation depends heavily on context, spatial resolution is not merely an add-on; it may be necessary to understand when ZDHHC9 is driving clinically relevant phenotypes and when it is not. This need is also consistent with broader recent reviews emphasizing the role of palmitoylation in immune modulation, metabolic rewiring, and treatment response.

### 6.5 Developing predictive biomarkers and rational patient stratification

Another major challenge is determining how ZDHHC9 can be translated into clinically useful biomarkers. Expression alone may not be sufficient. A tumor with high ZDHHC9 abundance may still differ dramatically from another ZDHHC9-high tumor if their substrate landscape, immune state, or depalmitoylation balance differs (77). For this reason, future biomarker development will likely need to combine several dimensions, such as ZDHHC9 expression, dominant downstream substrate status, checkpoint phenotype, and treatment context. Recent breast cancer studies are encouraging in that they associate ZDHHC9 with prognosis, tumor immunity, and potential immunotherapy relevance, but these analyses remain largely correlative. The next step is to test whether ZDHHC9-linked biomarker panels actually predict treatment benefit prospectively or in well-annotated retrospective cohorts.

### 6.6 Advancing precise therapeutic targeting of ZDHHC9-dependent palmitoylation

Perhaps the most translationally important future direction is the development of more precise interventions. At present, the therapeutic rationale for targeting ZDHHC9 is strong, but pharmacological tools remain limited. The pancreatic cancer study provides compelling proof of concept that tumor-directed ZDHHC9 silencing can enhance anti-PD-L1 therapy, while substrate-focused interference in the CD38 study shows that selective disruption of a single ZDHHC9-controlled palmitoylation event can also yield anti-tumor effects. More recently, emerging work has even reported candidate DHHC9 inhibitors in other cancer contexts, although these findings remain early and will require careful validation of selectivity and on-target action (78). Going forward, the most promising therapeutic strategies may include a combination of enzyme-selective inhibitors, degrader-style approaches, tumor-targeted RNA silencing, and substrate-interface blockers. The broader lesson is that “targeting ZDHHC9” should not be viewed as one single intervention, but as a family of possible strategies whose optimal form will depend on cancer type and dominant dependency.

### 6.7 Overall perspective

Taken together, the future of ZDHHC9 research will depend on moving from isolated mechanism papers toward an integrated framework that combines substrate mapping, tumor ecosystem analysis, biomarker development, and therapeutic precision. The field has already progressed beyond the stage of asking whether ZDHHC9 matters in cancer; the more pressing question now is when, where, and through which substrate networks ZDHHC9 matters most. Resolving that question will determine whether ZDHHC9 remains an intriguing mechanistic topic or develops into a clinically useful target at the interface of palmitoylation biology, tumor progression, and cancer immunity.

## 7 Conclusions

ZDHHC9 has emerged as an increasingly important palmitoyltransferase in cancer biology, linking reversible protein S-palmitoylation to multiple malignant phenotypes. Across diverse tumor types, current evidence indicates that ZDHHC9 can support tumor cell proliferation, survival, stress adaptation, oncogenic signaling, metabolic rewiring, and immune evasion through the palmitoylation-dependent regulation of selected substrates. At the same time, the available literature also makes clear that ZDHHC9 is not a uniform master regulator across all cancers; rather, its functions are best understood as context dependent, shaped by tumor lineage, substrate availability, signaling state, and microenvironmental conditions. A particularly notable aspect of ZDHHC9 is that it bridges tumor cell-intrinsic and immune-related biology. The ability of ZDHHC9 to influence checkpoint signaling, CD8+ T-cell activity, and response to anti-PD-L1 therapy suggests that its importance extends beyond classic oncogenic growth control and into the clinically consequential domain of tumor immune regulation. This dual role increases its translational appeal, especially in treatment-resistant tumors where malignant signaling and immune suppression coexist. Breast cancer biomarker analyses and pancreatic cancer functional studies together support the idea that ZDHHC9 may have value not only as a mechanistic target but also as a stratification marker for therapy-related decision making. Despite these advances, the field is still developing. Key unresolved issues include substrate specificity across cancers, PTM crosstalk, the spatial logic of ZDHHC9 activity in tumor ecosystems, and the lack of mature selective inhibitors. Future progress will require integrating modern acylation-mapping technologies with functional tumor immunology and translational drug development. With those advances, ZDHHC9 may become not just an emerging molecular player in cancer, but a mechanistically informed target for precision intervention.
